# Supplementary material for: Sentinel Lymph Node Biopsy in Surgical Staging for High-Risk Groups of Endometrial Carcinoma Patients
Source: Int J Environ Res Public Health. 2022 Mar 21;19(6):3716. doi: 10.3390/ijerph19063716 (PMC8949341; doi:10.3390/ijerph19063716)
Supplement: Supplementary file 1 [file ijerph-19-03716-s001.zip › Supplementary Table S1.pdf]

Supplementary Table S1. Characteristics of included studies and patients.

Study	Country	Setting	Study design	Time period	Patient selection	Eligibility criteria	Sample size			Age	BMI
							Total	High risk EC n (%)	Low risk EC n (%)	(Mean ± SD or median), [years +]	(Mean ± SD or median), [Kg/m²]
**2020 Cusimano**	Canada	University of Toronto; Odette Cancer Centre, Sunnybrook Health Sciences Centre; Trillium Health Partners, Mississauga	Multicentre prospective cohort study	Jul 2015 – Jun 2019	Consecutive	G2-G3 endometrioid, serous, carcinosarcoma, clear cell, undifferentiated or dedifferentiated, and mixed high grade clinically stage I.	156	156 (100)	0	65.5 (40 - 86)	27.5 (17.6 – 49.3)
**2019 Persson**	Sweden	Skåne University Hospital, Lund; Karolinska University Hospital, Stockholm	Multicentre prospective cohort study	Jun 2014 – May 2018	Consecutive	EC clinically stage I-II with at least one high-risk criteria (G3 endometrioid, non-endometrioid histology, 50% myometrial tumour invasion, cervical stromal invasion).	257	257 (100)	0	71 (44 - 90)	26.7 (17.3 – 47.3)
**2019 Ye**	China	Shanghai First Maternity and Infant Hospital	Prospective cohort study	Jul 2016 – Jul 2018	Consecutive	EC of any histology, clinically stage I or II. High risk histology: G3 endometrioid, carcinosarcoma, serous, clear cell, or undifferentiated carcinoma.	131	25 (19.1)	106 (80.9)	55.8 (35 - 76)	24.8 (18.5 – 37.8)
**2019 Wang**	China	Tianjin Central Obstetrics and Gynecology Hospital	Retrospective cohort study	Aug 2016 – Aug 2018	Consecutive	G1-G2 endometrioid with deep myometrial invasion or G3 endometrioid, serous, clear cell, or carcinosarcoma histology.	98	98 (100)	0	53 (37 - 69)	25.4 (21.2 – 33.5)
**2018 Papadia**	Switzerland	University Hospital of Berne	Retrospective cohort study	Dec 2012 – Jan 2018	Consecutive	Poorly differentiated endometrioid EC, uterine papillary serous carcinoma, clear cell carcinoma, neuroendocrine carcinoma, and carcinosarcoma.	42	42 (100)	0	65 (43 - 83)	26.8 (19 – 46.3)

-: not available; **EC:** endometrial cancer.
