# Supplementary material for: Sentinel Lymph Node Biopsy in Surgical Staging for High-Risk Groups of Endometrial Carcinoma Patients
Source: Int J Environ Res Public Health. 2022 Mar 21;19(6):3716. doi: 10.3390/ijerph19063716 (PMC8949341; doi:10.3390/ijerph19063716)
Supplement: Supplementary file 1 [file ijerph-19-03716-s001.zip › Supplementary Table S2.pdf]

Supplementary Table S2. Postoperative histotype and grade of endometrial carcinomas in the included studies.

Study	Postoperative histotype and grade								
	**Endometrioid, all grade, n (%)**	**Endometrioid, G1, n (%)**	**Endometrioid G2, n (%)**	**Endometrioid, G3, n (%)**	**Serous, n (%)**	**Carcinosarcoma, n (%)**	**Clear cell, n (%)**	**Undifferentiated, n (%)**	**Other, n (%)**
**2020 Cusimano**	65 (41.6)	0	30 (19.2)	35 (22.5)	52 (33.4)	17 (10.9)	3 (1.9)	5 (3.2)	14 (8.9)
**2019 Persson**	166 (64.5)	132 (51.4)		34 (13.3)	59 (22.9)	13 (5.0)	13 (5.0)	-	6 (2.3)
**2019 Ye**	6 (24.0)	0	0	6 (24.0)	12 (48.0)	3 (12.0)	4 (16.0)	-	-
**2019 Wang**	72 (73.5)	43 (43.9)		29 (29.6)	10 (10.2)	-	12 (12.2)	4 (4.1)	-
**2018 Papadia**	24 (57.1)	-	-	-	9 (21.4)	5 (11.9)	3 (7.1)	-	1 (2.4)
**Total**	**333 (57.6)**	**-**	**-**	**-**	**142 (24.6)**	**38 (6.6)**	**35 (6.1)**	**9 (1.6)**	**21 (3.5)**

-: not available.
