# Supplementary material for: Sentinel Lymph Node Biopsy in Surgical Staging for High-Risk Groups of Endometrial Carcinoma Patients
Source: Int J Environ Res Public Health. 2022 Mar 21;19(6):3716. doi: 10.3390/ijerph19063716 (PMC8949341; doi:10.3390/ijerph19063716)
Supplement: Supplementary file 1 [file ijerph-19-03716-s001.zip › Supplementary Table S3.pdf]

**Supplementary Table S3.** Postoperative FIGO stage of endometrial carcinomas in the included studies.

| Study | Postoperative FIGO stage n (%) | | | | | | | | |
|---|---|---|---|---|---|---|---|---|---|
| | **IA** | **IB** | **II** | **IIIA** | **IIIB** | **IIIC** | **IIIC1** | **IIIC2** | **IV** |
| **2020 Cusimano** | 93 (59.6) | 20 (12.8) | 12 (7.7) | 3 (1.9) | - | 27 (17.4) | 19 (12.2) | 8 (5.2) | 1 (0.6) |
| **2019 Persson** | 37 (14.4) | 143 (55.6) | 13 (5.1) | - | 3 (1.2) | 56 (21.8) | 33 (12.8) | 23 (8.9) | 5 (1.9) |
| **2019 Ye** | 96 (73.3) | 18 (13.7) | 4 (3.1) | 5 (3.8) | - | 8 (6.2) | 4 (3.1) | 4 (3.1) | - |
| **2019 Wang** | 37 (37.6) | 39 (40.0) | - | - | - | 22 (20.6) | 20 (20.4) | 2 (2.0) | - |
| **2018 Papadia** | 17 (40.5) | 11 (26.2) | 1 (2.4) | 3 (7.1) | - | 10 (23.8) | 2 (4.8) | 8 (19) | - |
| **Total** | **280 (40.9)** | **231 (33.8)** | **30 (4.4)** | **11 (1.6)** | **3 (0.4)** | **123 (18.0)** | **78 (11.4)** | **45 (6.6)** | **6 (0.9)** |

**FIGO:** International Federation of Gynecology and Obstetrics; **-:** not available**.**
