# Supplementary material for: Sentinel Lymph Node Biopsy in Surgical Staging for High-Risk Groups of Endometrial Carcinoma Patients
Source: Int J Environ Res Public Health. 2022 Mar 21;19(6):3716. doi: 10.3390/ijerph19063716 (PMC8949341; doi:10.3390/ijerph19063716)
Supplement: Supplementary file 1 [file ijerph-19-03716-s001.zip › Supplementary Table S4.pdf]

Supplementary Table S4. Details about sentinel lymph node biopsy.

Study	Route of surgery	ICG injection technique				Pathology assessment
		Concentration [mg/mL]	Dose [mg]	Site	Deepness [mm]	
**2020 Cusimano**	Laparoscopy or Robotic	2.5	5	3 and 9 h	1-2 and 10	H&E, IHC ultrastaging
**2019 Persson**	Robotic	2.5	2.5 +/- 2.5 (after 10 min in case of non mapping)	2, 4, 8 and 10 h +/- 3 and 9 (after 10 min in case of non mapping)	Submucosally and 30	H&E, IHC ultrastaging
**2019 Ye**	Laparoscopy	0.5	2	3 and 9 h	Superficially and deeply	H&E, IHC ultrastaging
**2019 Wang**	-	-	-	3 and 9 h	1-3 and 10	H&E, IHC
**2018 Papadia**	Laparoscopy	5 or 2.5 since 2017	40 or 20 since 2017	3, 6, 9, 12 h	10	H&E, ultrastaging

-: not available; **ICG:** Indocyanine Green; **H&E:** Hematoxylin and eosin stain; **IHC:** immunohistochemistry.
