# Supplementary material for: Sentinel Lymph Node Biopsy in Surgical Staging for High-Risk Groups of Endometrial Carcinoma Patients
Source: Int J Environ Res Public Health. 2022 Mar 21;19(6):3716. doi: 10.3390/ijerph19063716 (PMC8949341; doi:10.3390/ijerph19063716)
Supplement: Supplementary file 1 [file ijerph-19-03716-s001.zip › Supplementary Table S5.pdf]

Supplementary Table S5. Details about sentinel lymph node biopsy and systematic lymphadenectomy.

Study	P LND n (%)	PPA LND n (%)	Successful mapping of SLN n (%)	Bilateral mapping of SLN n (%)	PA SLN Detected n (%)	n of SLN detected n (%)	PA LN Metastases n (%)*	Isolated PA metastases n (%)*	n of LN removed		Disease limited to the SLN n (%)**
									Pelvic (Mean ± SD or median)	Para-aortic (Mean ± SD or median)	
**2020 Cusimano**	156 (100)	101 (64.7)	152 (97.4)	121 (77.6)	-	3 (2-5)	8 (7.9)	-	16 (12-20)	5 (3-9)	14/27 (51.8)
**2019 Persson**	257 (100)	217 (84.4)	247 (96.1) and 254 (98.8) after reinjection	212 (82.4) and 241 (93.8) after reinjection	-	4 (1-7)	23 (10.6)	2 (0.9)	29 (8-75)	12 (2-51)	28/54 (51.8)
**2019 Ye**	131 (100)	25 (19)	122 (93.1)	81 (61.8)	4 (1.3)	2 (1 - 7)	4 (16)	3 (12)	28 (12 - 60)	10 (2 - 18)	3/8 (37.5)
**2019 Wang**	98 (100)	-	94 (95.9)	76 (77.6)	2 (2)	1.8 (0 - 6) and 1.2 (0 - 3)***	2 (-)	-	23 (11 - 40)	14 (5 - 26)	10/22 (45.5)
**2018 Papadia**	42 (100)	42 (100)	42 (100)	38 (90.5)	-	3 (1 - 18)	8 (19)	3 (7.1)	54 (21 - 83)		-

LND: systematic lymphadenectomy; **P:** Pelvic; **PA:** Para-aortic; **SLN:** Sentinel lymph node; **LN:** lymph node; -: not available.
***:** Data regarded patients who underwent PA LND.
****:** Data regarded patients with node-positive disease.
*****:** on the right and left side respectively.
